# Porous Titanium Granules and Blood for Bone Regeneration around Dental Implants: Report of Four Cases and Review of the Literature

**DOI:** 10.1155/2013/410515

**Published:** 2013-03-07

**Authors:** Andreas Thor

**Affiliations:** Department of Plastic & Oral and Maxillofacial Surgery, Institute of Surgical Sciences, Uppsala University, 751 85 Uppsala, Sweden

## Abstract

A regenerative procedure treating a local osseous defect around titanium dental implant using porous titanium granules is described in four patients. Porous titanium granules represent, for maxillofacial surgery, a new alternative in augmenting osseous defects. Its earliest application was in the field of orthopedics for stabilization of tibia plateau fractures and for reoperations in prosthetic fixation of femoral stems. There is emerging scientific evidence regarding titanium for its potential use in the maxillofacial area and porous titanium granules are now commercially available. The scientific background for the osteoconductive use of porous titanium granules is elucidated in this paper and the supporting literature is reviewed.

## 1. Introduction

Bone augmentation of the alveolar crest in implant dentistry has attracted a substantial interest in the maxillofacial literature [1, 2]. Bone substitutes represent an important contribution to this field as augmentation with autogenous bone has drawbacks such as morbidity for the patient as well as problems with various degrees of resorption of the grafted bone volume over time [3].

After installation of dental implants, multifactorial conditions may all contribute to a peri-implant state of disease, for example, poor oral hygiene and a history of periodontitis, diabetes, or smoking. The mucosal status of attached or free gingiva at the fixture site is also of importance for long time success [4]. This problem has recently been discussed extensively in the dental implant literature; soft tissue peri-implant mucositis may affect about 50% of the implant sites and bone-affecting peri-implantitis may be seen in as many as 12–40% of the implant sites according to a consensus report from a European group of workers [4]. Implantology offers huge possibilities for patients and the restorative team, but the potential problem that may arise around implants needs to be addressed and not neglected by the implant surgeon. 

The candidates among bone substitutes have been many over the years and some have proven to be successful [5]. Porous titanium granule (PTG) represents a new alternative in augmenting osseous defects in maxillofacial surgery. Its earliest application was seen in orthopedics and used for stabilization of tibial plateau fractures and for prosthetic reoperations for femoral stem fixations. A case operated in 1995 where dental implants in a split-crest procedure were supported by PTG represents the earliest reported surgery in the literature [6–9]. There is emerging scientific evidence of this material regarding the potential use in the maxillofacial area and the material is now marketed commercially [10, 11]. The aim of this paper is to describe a procedure treating local osseous defects around titanium dental implants using porous titanium granules in four patients. The available literature, compiled through a search on PubMed, is also discussed.

## 2. Case Presentation

**Figure 1:**
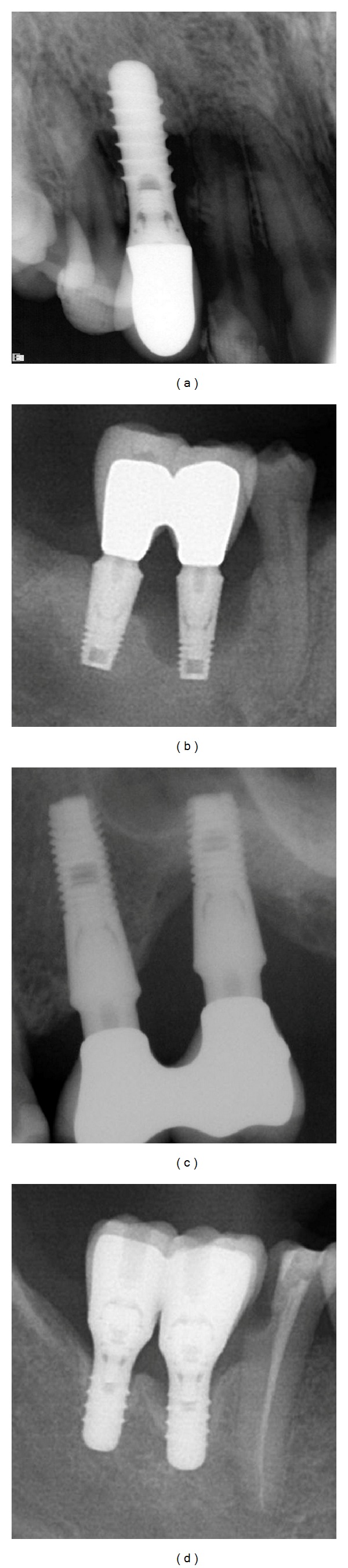
Preoperative radiological situation in subjects 1–4.

Four patients, three females and one male, were scheduled for peri-implant regenerative surgery. All presented with local defects around implants some time after prosthetic loading of the implants (Table 1). The male patient previously went through a periodontal surgical debridement for removal of granulation tissue around the affected two implants in the lower right premolar area, but bone destruction around the implants progressed. All four patients were preoperatively evaluated with clinical and radiological examination (Figure 1). Prosthetic overload was suspected as a contributing factor in three patients due to localized defects around implants and the occlusion was corrected accordingly in these patients prior to surgery. In two of the cases the defects were extensive and the procedure with PTG was considered as a salvage therapy for the implants that only displayed osseointegration in the most apical part. 

**Figure 2:**
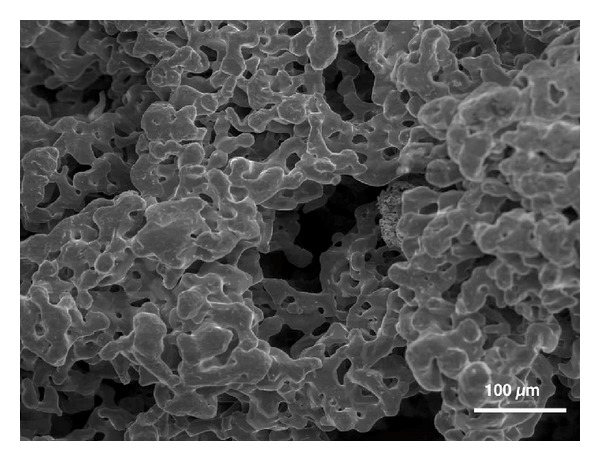
A porous titanium granule (PTG) seen with SEM. Particle size 500–1000 *μ*m with a total porosity of approximately 80%.

**Figure 3:**
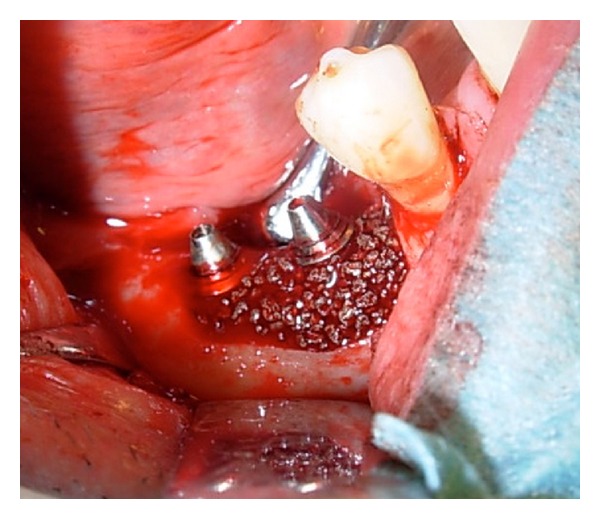
Clinical example of PTG implanted in marked vertical defect around implants in the lower right jaw of patient number 2.

**Figure 4:**
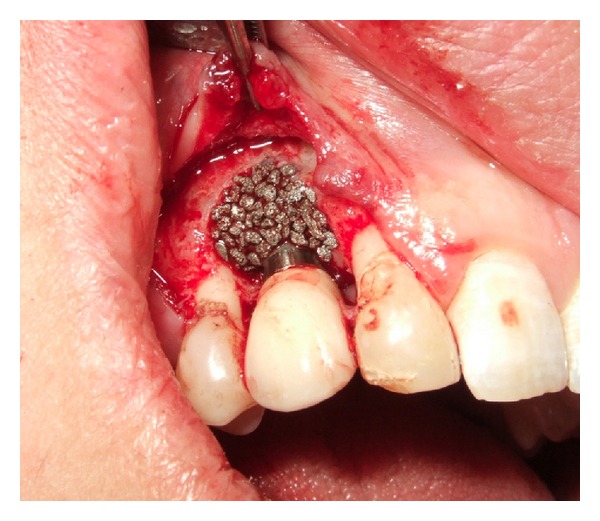
Clinical example of PTG implanted in bone defect around implant in position of upper right canine of patient number 1.

Antibiotics were administered preoperatively (2-gram phenoxymethylpenicillin, Kåvepenin, Astra Zeneca, Södertälje, Sweden, and metronidazole, Flagyl, Sanofi-Aventis, Bromma, Sweden). Surgery was commenced in local anesthesia as flaps were raised in the area of the affected implants in order to augment the defects. Debridement of the surgical sites was performed. Granulation tissue in the defects as well as on the flaps was removed. After carefully cleaning the implants with scalers as well with a titanium brush (Tigran Brush No1, Tigran Technologies, Malmö, Sweden), rinsing of the surgical site with 3% hydrogen peroxide (H_2_O_2_) complemented by copious amounts of saline (NaCl), porous titanium granules (Tigran Technologies, Malmö, Sweden) were inserted in the defects and mixed with blood. The bony walls of the defects were prepared to stimulate small bleeding points with a small bur prior to insertion of the granules. Following the protocol supplied by the manufacturer, PTG (particle size 700–1000 *μ*m, Figure 2) was gently condensed into the defects and around the fixtures up to the superior part of the implant, filling out the defects. In the three patients where overload of the implant retained bridge was suspected and the occlusion corrected preoperatively, the supraconstructions were temporarily removed during surgery for access to the surgical site and then replaced after suturing. The level of augmentation mimicked the original previous bone level at the installation of the fixtures (Figure 3). The granules connected well together in the clot forming a lightly moldable mass of the augmentative material. After the removal of visible granulation tissuefromthe flaps, they were repositioned with nonresorbable sutures around the restorations (the two-unit bridge in the three patients was repositioned at this time). In the fourth patient, who had a localized defect probably caused by a remaining minor tooth fragment in close contact with the installed implant eventually causing a defect in the bone, PTG was also densely inserted into the defect (Figure 4) and the flap repositioned accordingly. Antibiotics (metronidazole starting preoperatively 3 days for the male patient due to a severe local bony destruction and pus formation around one of the fixtures; phenoxymethylpenicillin for the three female patients) were administered also postoperatively for all patients for one week. Chlorhexidine mouth rinse was used for the patients for two weeks postoperatively. Patients were advised not to clean the operated area mechanically with a brush for the first 10 days after augmentation. Sutures were removed one week postoperatively and all patients tolerated the procedure well with no adverse reactions recorded. Patients have now been followed between 9 to 26 months.

**Figure 5:**
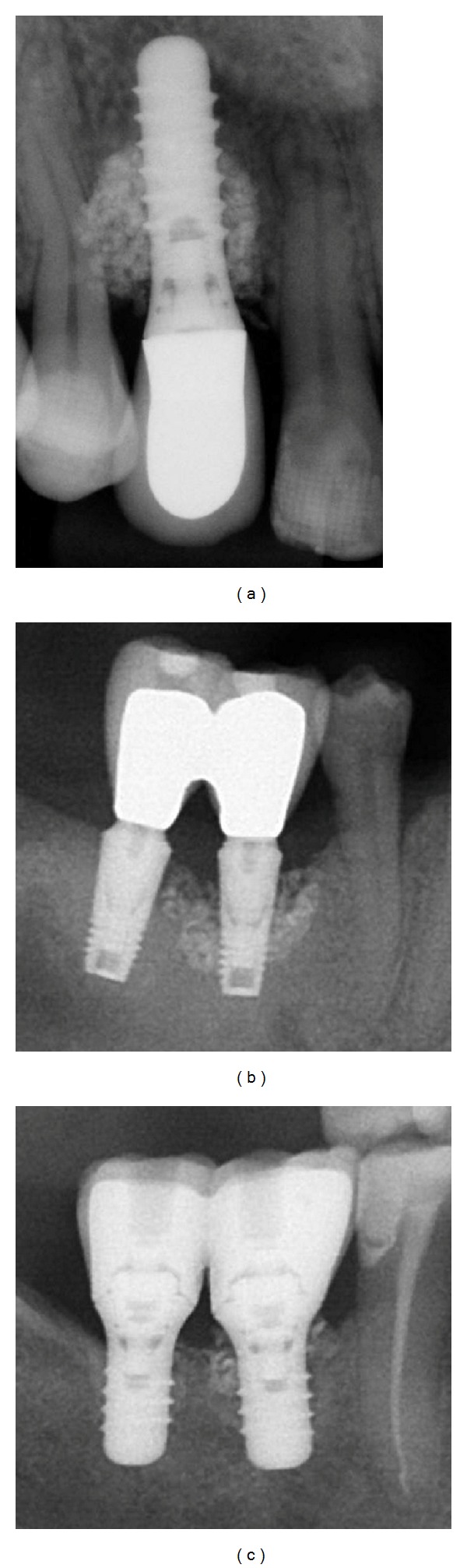
Radiological followup of patients 1, 2, and 4 after 26, 12, and 9 months, respectively, after surgery.

**Figure 6:**
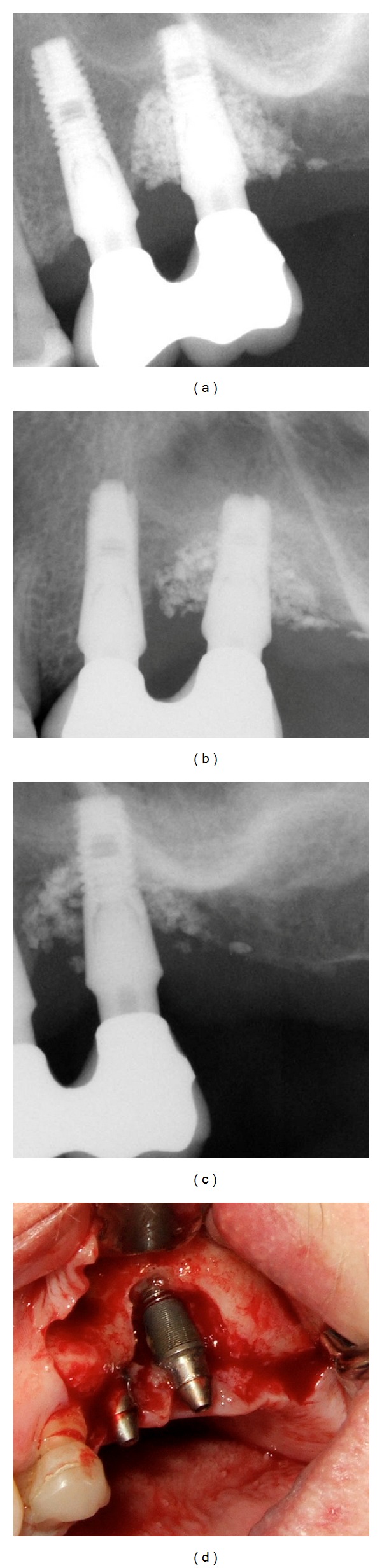
Illustration of sequence of radiological followup and clinical baseline status of patient number 3. Note the marked loss of grafting material in the defect (narrow crest). Extensive filling of defect with PTG at surgery resulted in loss of grafting material but with a gingival level that accordingly was positioned more apically without signs of marked inflammation or infection.

Clinical evaluation displayed a stable situation around the implant in two of the four patients with no loss of grafting material during followup. In the other two patients, the level of the marginal gingiva had been apically positioned and the loss of grafting material from the site of augmentation was progressively seen during the first month of followup, also reflected in the radiological examinations on followup (Figures 5 and 6). However, no visible inflammation or pus formation nor bleeding was seen around these implants. None of the implants have been lost in the followup and progression of bone loss appears to be halted.

## 3. Discussion

This paper reports on the results of four patients illustrating a rather novel technique for bone augmentation in the maxillofacial area using PTG. However not yet extensively explored regarding its use the biomaterial renders interest and is supported on the basis of innovative ideas that may be clinically important in the future. Bone substitutes represent an important area in reconstructive dental implantology and have been studied extensively [3, 12, 13]. The search for osteoinductive as well as osteoconductive materials has lead to the novel idea of using titanium in bone augmentations of the alveolar crest [10, 14]. The indications for the use of PTG in this area have so far been limited to recently reported sinus augmentations and for defects around dental implants, even though the idea was first tried in a pilot case in 1995 [6, 11]. Just recently, Wohlfahrt et al. published a paper evaluating PTG in mandibular degree II furcation defects. The authors considered the material as safe in close proximity of root surface but found that the clinical result of the surgical treatment with PTG in 10 patients was diverging regarding the studied parameters. The values of probing depth and radiographic vertical furcation height were significantly reduced after one year, but no significant results were seen in clinical attachment levels or gingival recession [15]. Additionally, a clinical study where defects aroud implants were treated with PTG, surgical debridement, decontamination, and healing in a submerged fashion for six months, showed radiological improvement around implants but no significantly different clinical improvement compared to the same treatment without the use of PTG [16].

The overall experimental and clinical extent of the available literature so far is limited. PTG was initially used in orthopedics for stabilization of tibial plateau fractures and for reoperations in prosthetic fixation of femoral stems. Clinical results in a limited amount of patients in two case series reported satisfactory results on the materials ability to form a stable foundation for femoral stems and support for the elevation of condylar bone in depressed proximal tibia fractures [7, 8]. The material has also been evaluated in a clinical study of sinus augmentations where the main part of the included study subjects (12 patients) had implants simultaneously installed and PTG placed around them in the sinus floor in a one-stage procedure. Four patients had a delayed placement of implants due to insufficient primary stability at the time of augmentation. Three implant losses after an observation period of 12–36 months were seen and two of these were in the staged group. In the simultaneous placement group one implant was lost after one year of loading (after a history of postoperative sinus infection). The authors raised questions regarding the usage of the material in staged sinus lifts as well as further explore the risk of displacement of granules into the sinus during augmentation [11]. 

PTG has been subject to comparison with other grafting materials. A recent in vitro study with human primary mesenchymal stem cells comparing PTG with bovine bone (Bio-Oss, Geistlich Pharma AG, Wolhusen, Switzerland) and *β*-tricalcium phosphate (Bone Ceramic, Straumann AG, Basel Switzerland) reported better results for PTG regarding cell viability and proliferation as well as porosity, interconnectivity, open pore size, and surface area-to-volume ratio [17]. Additionally, Faria et al. explored the use of titanium in filling bone defects in a canine model with the purpose of comparing porous *titanium sponge rods* with synthetic hydroxyapatite. The titanium foam was constructed by mixing together titanium powder and space-holding particles and thereafter compacted and sintered: first to remove the space holding particles resulting in giving the titanium powder a porous structure [18]. In the humerus of six mongrel dogs, a total of 36 defects were randomly either filled with titanium foam in the shape of rods, particulate hydroxyapatite, or a blood clot serving as control. Healing times of two or four months were used and results displayed a higher density of grafting material in the test defects with titanium foam and less amount of newly formed bone accordingly compared to control defects with coagulum. Titanium foam and hydroxyapatite were comparable regarding new bone formation in both time intervals. Interestingly, the authors stated that the better contact between the marginal bone and the titanium material resulted in a better preservation of the marginal bone levels in the defects filled with titanium-foam compared to control. More mature bone was also seen around the titanium material. The titanium-foam, with a porosity of about 80% (same as in PTG used in the presented cases) and with good ingrowth properties for bone, was proposed as material for future use in bone augmentation [14].

One explanation for successfully maintaining the marginal bone levels in the defects in the previously mentioned study and for swift maturation of new bone may be the use of titanium in contact with whole blood which has been discussed and proposed in the literature as a relevant combination of materials for bony reconstruction [19, 20]. Blood can be regarded as the primary tissue in “gap” bony healing, as seen in fractures [21], distraction osteogenesis [22], or even in the maxillary sinus floor when using the sinus elevation technique for simultaneous installation of implants without the use of bone grafts or bone substitutes [23, 24]. However, initially the search for a biocompatible material to be used in the human vascular system or for blood contact medical devices (e.g., stents, vascular grafts and catheters) led to the recommendation of avoiding the use of titanium, as the material was found to be very potent regarding activation of the coagulation system with unwanted thrombus formation as a result of blood contact. Thrombus formation and inflammation involve the activation of both the coagulation and the complement system: the initiation of these systems causes plasma protein adsorption on the surface followed by the activation and adhesion of platelets and leukocytes [25]. The use of titanium as well as tantalum and indium in an in vitro study was found to display pronounced thrombogenic properties. Aluminium, nickel, and especially iridium were regarded as nonthrombogenic [26]. The opposite potential positive effect for the use of titanium in bone was therefore introduced, where the thrombogenic activity of the material could be useful for the onset of a rapid start of the healing process in healing of bone [23, 27]. 

Modifications of the titanium surface of a dental implant regarding topography, structure, surface chemistry, charge, and wettability have been proven to alter the osseointegration [28]. As an example, a surface modification of titanium that was titanium dioxide blasted and etched with hydrofluoric acid displayed the ability to enhance the onset of coagulation and caused a stronger release of growth factors in contact with blood [20]. Another titanium surface was chemically modified (hydroxylated/hydrated) resulting in increased hydrophilic properties and displayed superior results compared to control smooth titanium surfaces regarding influence on cell differentiation and growth factor release as well as improving soft and hard tissue integration [29]. Recently, a lot of focus has been directed to the fact that surface modifications result in a nanotopography of the implant surface and investigations continue to be published in the literature. Nanotopography in the form of TiO_2_ nanotubes resulted experimentally in better proliferation and adhesion of osteoblasts resulting and improved bone-bonding strength as compared to TiO_2_-gritblasted surfaces [30]. The importance of this nanotexture was confirmed as nanoporous alumina membranes with pore diameters of 20 and 200 nm were shown to activate platelets differently. Platelet microparticle generation, as a marker of procoagulant activity, was shown to be higher in pore size of 200 nm. As smooth alumina earlier was shown to be nonthrombogenic, these studies indicate that there may be a direct influence on thrombogenicity from the size of nanopores. The optimal pore size is still to be explored [26, 31, 32]. Smooth, machined titanium as well as modified titanium surfaces has proven to be efficient in the activation of the coagulation cascade in blood contact and one may therefore argue its value as a material used for regeneration in defects with new bone formation. Furthermore, the porous titanium granules are hollow in their total volume to 80 percent. The capillary action on blood to fill these pores in the granules parallel to the supposed strong initiation of coagulation and release of growth factors and clot formation results clinically in the formation of a moldable mass that can be placed in peri-implant defects. The results of the cases presented in this paper show however that the use must be with moderation; bone formation outside the skeletal envelope, or as in these two cases reported herein where loss of grafting material was rather extensive, is not easily achieved. The affected *implants *were not covered by the flaps after the defects were augmented with the scaffold of PTG. Submerging the implants may have resulted in a better outcome and less loss of material in two of the four cases where the initial crest was narrow. Coverage of the graft with a membrane may also increase the level of outcome. Overloaded supraconstructions may have added to the bony defect progression around the implants in these two specific cases. The reasons for progression for defects around implants must be widely investigated. Another issue is the availability of attached gingival around implants, something that may be a commonly encountered problem in situations of long-standing edentulism, for example, in the lower jaw premolar area. 

In conclusion, further clinical and experimental studies on the use of porous titanium granules as a bone augmentation material are needed, even though there are some studies published on the material to support its use.

## Figures and Tables

**Table 1:** Demographic data describing the four study subjects.

Subject	Gender	Age	Implant position	Time of preop. prosthetic loading	Duration of followup
1	F	37	Upper right canine	12 months	26 months
2	M	58	Lower right premolar	36 months	12 months
3	F	66	Upper right molar	84 months	11 months
4	F	64	Lower right molar	22 months	9 months
